# Daily Empowering Leadership and Job Crafting: Examining Moderators

**DOI:** 10.3390/ijerph17165756

**Published:** 2020-08-09

**Authors:** Shangbiao Tang, Guanglei Zhang, Hai-Jiang Wang

**Affiliations:** 1 School of Economics and Management, Southwest Jiaotong University, Chengdu 610000, China; tangshangbiao@163.com; 2 School of Management, Wuhan University of Technology, Wuhan 430000, China; 3 School of Management, Huazhong University of Science and Technology, Wuhan 430000, China; hjiangwang@gmail.com

**Keywords:** daily diary, leader empowering behaviors, proactive motivation, job crafting, work meaning, vigor

## Abstract

In this study, we built and tested a contingency model linking leader daily empowering behaviors with employee daily job crafting. Drawing on the contingency leadership literature and the model of proactive motivation, we theorized employee daily work meaning and vigor as moderators of the above relationships. Daily data were collected from 103 Chinese employees for five consecutive days. Our findings suggest that leader day_T_ (a certain day) empowering behaviors are more strongly related to employee day_T+1_ (next day after the certain day) job crafting when employee day_T_ work meaning is low and employee day_T+1_ vigor is high. Our findings suggest that only under certain conditions can empowering leadership promote employee job crafting on a daily basis.

## 1. Introduction

It has been well recognized that employee motivation and health are affected by work design [1]. Researchers and practitioners have thus devoted considerable effort in understanding how to design the work to increase work motivation and optimize employee functioning. Job crafting was defined as “the physical and cognitive changes individuals make in the task or relational boundaries of their work” [2]. As a bottom-up approach to work design, job crafting has recently attracted much attention [3] Researchers suggest that one noticeable way to empower employees to job craft is through leadership. Empowering leadership characterized by delegating authority and autonomy to employees [4] may make employees feel that they can change the work design. Although job crafting researchers have established linkages between leadership and employee job crafting [5,6,7,8,9,10,11,12], little is known about boundary conditions for the effect of empowering leadership, in particular, on job crafting. 

Empowering leadership may make employees feel empowered, but it does not necessarily guarantee that they will change the work design. We argue that the relationship between empowering leadership and job crafting is likely to be contingent upon other personal and job factors [3]. According to the model of proactive motivation [13], proactive behavior such as job crafting takes effort and is inherently uncertain and risky; the motivation underlying employee proactive behavior consists of multiple components such as “can do”, “reason to”, and “energized to”. Working meaning refers to the extent to which job incumbents experience their job as meaningful, valuable, and worthwhile, which may vary significantly from day-to-day [14]. Vigor refers to high levels of energy while working [15]. We suggest that leader empowerment (enabling “can do” motivation) may be more effective in promoting employees’ job crafting when their work meaning is low (“reason to” motivation) and their vigor is high (“energized to” motivation). Thus, we propose that empowering leadership may interact with work meaning and vigor in predicting job crafting. In doing so, our study contributes to the literature in three major ways. 

First, job crafting is self-initiated behavior that is aimed to mold and shape the design of the job (e.g., job demands and resources) to make it more meaningful and motivating. Given greater competition and increased demand for innovation, employees are needed and encouraged to shape their work roles in the job by crafting what they do and how they do it in order to better serve organizational goals [16,17]. That is, employees are empowered and placed in a position of designing the job, which is traditionally held by managers or leaders. It is thus important to understand whether such empowering effort by leaders can increase job crafting among employees. By adopting a contingency perspective our study provides a more nuanced understanding of when empowering leadership increases job crafting, adding to the growing literature on leadership and employee job crafting. 

Second, we enrich the contingency perspective on empowering leadership and employee outcomes by theorizing boundary conditions based on the model of proactive motivation. According to Parker et al. [13], rather than one motivational state, proactive behavior (job crafting) is sparked by multiple motivational states. “Can do” motivation arises from a sense of control and efficacy; “reason to” motivation refers to the extent to which an individual has compelling reason to be proactive; “energized to” motivation refers to activated positive affect that stimulates proactive behavior. Although it increases one’s “can do” motivation, empowering leadership alone may not necessarily guarantee job crafting behavior. In other words, even if employees are under empowering conditions, they may have no compelling reason or energy to craft their job. However, previous studies examining (empowering) leadership as an antecedent of job crafting have not simultaneously considered other necessary motives for job crafting. Addressing this gap, our study examines the three motives for job crafting and shows that how they interact with one another in predicting job crafting. 

Third, beyond the between-person studies, we focus on day-level empowering leadership as an antecedent of day-level job crafting and attempt to gain more insight into how short-term, within-person fluctuations in leader empowering efforts relate to daily job crafting. The daily diary design is suitable for testing our model. Job crafting research acknowledges “the everyday altering of jobs that individuals do”; that is, “the job is being re-created or crafted all the time” [2]. It is thus critical to understand the dynamics of job crafting behavior. Although empowering leadership has been viewed as a leadership style involving delegation of power, it may fluctuate on a daily basis (within-person) depending on various situational factors [18]. Likewise, employees may experience work meaning [14] and vigor [19] on some days, but not on others, due to, for example, daily importance of work tasks, social interactions, and sleep quality. 

Adopting a within-person approach is critical to understand how job crafting is dynamically influenced by empowering leadership, work meaning, and vigor. It is reasonable to suggest that leaders may adapt their leadership behaviors to the situational conditions of employees [20,21]. For a particular employee, he or she might need more (or less) leader empowerment on some days than others, leaders may adjust the level of their empowering efforts accordingly. Besides, depending on a leader’s own job factors (e.g., workload) and personal factors (e.g., sleep quality), a leader’s behavior may fluctuate on a daily basis [22], which may in turn influence employee job crafting. 

We organize the present paper as follows. First, we present the theoretical background of job crafting and empowering leadership. Next, we develop our hypotheses mainly based on the proactive motivation model. Then, we report the results of a daily diary study. Finally, we discuss the theoretical and practical implications and limitations of our study, followed by a short conclusion.

## 2. Theoretical Background and Hypotheses

### 2.1. Employees as Active Crafters of Their Own Jobs

To better capture what changes job crafters make to the job, European scholars defined job crafting using the framework of the Job Demands-Resources (JD-R) theory [23,24]. The JD-R theory classifies job characteristics into two categories: job demands and job resources [25,26]. Job demands are primarily associated with stress and job resources are primarily associated with motivation. Job crafting was viewed as “changes that employees initiate in the level of job demands and job resources in order to make their own job more meaningful, engaging, and satisfying” [27], consisting of seeking resources, seeking challenges, and reducing demands [23]. Seeking challenges (e.g., asking for more tasks or responsibilities) and reducing demands (e.g., diminishing emotional, cognitive, or physical job demands) can be seen as altering task boundaries, while seeking resources (e.g., contacting other people at work to get work-related information) can be seen as altering relational boundaries. 

### 2.2. Leadership and Employee Job Crafting 

Recently, researchers have begun to empirically examine the linkages between leadership and job crafting. Servant leadership, a people-oriented leadership style, has been found to increase employees’ job crafting. For example, Harju et al. [6] found that team-level servant leadership predicted less job boredom by boosting job crafting. Bavik et al. [5] found that servant leadership promoted employees’ prosocial behavior by boosting job crafting. Moreover, Lichtenthaler and Fischbach [9] found that people-oriented leadership was positively related to expansion-oriented job crafting but unrelated to contraction-oriented job crafting. Transformational leadership, a change-oriented leadership style, may also matter to job crafting. Supporting this notion, Hetland et al. [7] found that employees’ day-level perception of their leader’s transformational behavior was positively related to employees’ day-level job crafting in the form of increasing structural and social resources. The study by Wang et al. [12] further suggested that transformational leadership may promote employees’ expansion-oriented job crafting by increasing their adaptability. There has also been some evidence for the relationship between empowering leadership and job crafting. Kim and Beehr [8] found that after controlling for proactive personality empowering leadership still related to employees’ job crafting. The study by Thun and Bakker [11] further suggested that the association between empowering leadership and (expansion) job crafting may be stronger for employees who are more optimistic. Employees with high leader-member exchange (LMX) are more trusted and supported by supervisors and therefore have more autonomy and freedom to craft their jobs. Finally, from a relational perspective, Radstaak and Hennes [10] found that high quality of leader–member exchange may be helpful for employees to engage in job crafting. 

From the above brief review, we suggest that there has been accumulating evidence showing the linkages between leadership and employee job crafting. Yet, we still know little about when empowering leadership in particular is more or less effective in stimulating job crafting on a daily basis. This is an important gap because contingency leadership theories suggest that to what extent leaders’ empowering efforts is effective is dependent upon employees’ personal and job characteristics [28]. Moreover, researchers suggest that leader empowerment varies not only between employees but within employees [18]. That is, different employees may receive more or less empowerment from the leader and a particular employee may receive more empowerment on some days, but less on the other days. 

### 2.3. Leader Daily Empowering Behaviors

There have been two approaches to the research on empowerment. The first one defines empowerment as a set of organizational practices mainly involving delegation of responsibilities and authority so that decisions with respect to the execution of work tasks can be made at a lower hierarchical level [29]. The second one conceptualizes empowerment as a psychological state characterized by perceptions of meaningfulness, competence, self-determination, and impact [30]. Following previous studies [31,32] and in line with the first approach, we define empowering leadership as leader behaviors that include the following four dimensions: consulting (e.g., listening to employees’ ideas and concerns before making a decision), delegating (e.g., increasing employees’ responsibilities and authority), enabling (e.g., expressing confidence in employees’ ability to perform at a high level), and informing (e.g., explaining how employees’ work relates to objectives and goals of the company). Empowering leadership has been found to enhance employees’ psychological state of empowerment [31,32,33,34]. 

According to the contingency theories of leadership, empowering leadership may not always fit to a particular situation [17,35]. Researchers suggest that the effectiveness of empowering leadership is dependent on characteristics of employees and contexts where they work [31,32]. Drawing from the model of proactive motivation [13], we further theorize in the next sections that employee daily work meaning and vigor may jointly interact with leader daily empowering leadership in promoting employee daily job crafting. 

### 2.4. Daily Empowering Leadership and Job Crafting: The Moderating Role of Employee Daily Work Meaning and Daily Vigor

We suggest that leader empowerment may stimulate employee job crafting on the days that employees experience low work meaning. Working meaning may vary significantly from day-to-day [14]. We argue that leader daily empowering behaviors may have a stronger relationship with employee daily job crafting when employee daily work meaning is low. This is consistent with the notion that the effectiveness of a specific type of leadership behavior is dependent upon the needs of a particular situation. When employees are faced with little work meaning, they may have a stronger need for change to the work design (“reason to” motivation) because pursuing meaning is a fundamental motivator of employee behavior [36,37]. Leader empowering behaviors such as delegation of authority may unlock the constraints of rules and regulations so that employees can shape their jobs into those more personally meaningful to them [38]. By contrast, since a highly meaningful work situation is satisfying and motivating in itself, employees would not have the need and reason to craft their job. Thus, leader empowerment may not spark employee job crafting when employees experience high levels of meaning. In other words, we propose that work meaning may attenuate the effect of empowering leadership on job crafting. Based on the above, the following hypotheses are formulated:

**Hypothesis** **1 (H1).** *Daily work meaning will negatively moderate the relationships between leader daily empowering behaviors and employee daily seeking resources (H1a), daily seeking challenges (H1b), and daily reducing demands (H1c).*

We suggest that leader empowerment may stimulate employee job crafting on the days that employees experience high vigor. As vigor is influenced by recovery experiences prior to work, sleeping quality, and emotions which are likely to change from day to day, vigor also fluctuates heavily on a daily basis [19]. Job crafting as a change-oriented behavior takes new effort, which may particularly require employees to be vigorous (“energized to” motivation). On days that employees perceive low vigor they do not have the energy to make change to the job, even they are provided with autonomy and authority by leaders. Leader empowerment is somewhat not effective in this situation. In contrast, vigorous employees can better “take the ball and run with it” working with an empowering leader. Therefore, vigor may strengthen the effect of empowering leadership on job crafting. Based on the above, the following hypotheses are formulated:

**Hypothesis** **2 (H2).** *Daily vigor will positively moderate the relationships between leader daily empowering behaviors and employee daily seeking resources (H2a), daily seeking challenges (H2b), and daily reducing demands (H2c).*

Further, we argue that low work meaning together with high vigor may result in job crafting to a great extent in the context of empowering leadership (joint moderating effects) [39]. According to the model of proactive motivation, proactive behavior is triggered by motives where employees feel able to, have a reason to, and are energized to initiate proactivity. We suggest that these three motives may combine and interact with one another to predict employee proactive behavior. Accordingly, we propose a three-way interaction of empowering leadership, work meaning, and vigor in predicting employee job crafting. Additionally, we present our hypotheses as follows:

**Hypothesis** **3 (H3).** *Daily work meaning and daily vigor will jointly moderate the relationships between leader daily empowering behaviors and employee daily seeking resources (H3a), daily seeking challenges (H3b), and daily reducing demands (H3c).*

**Figure 1:**
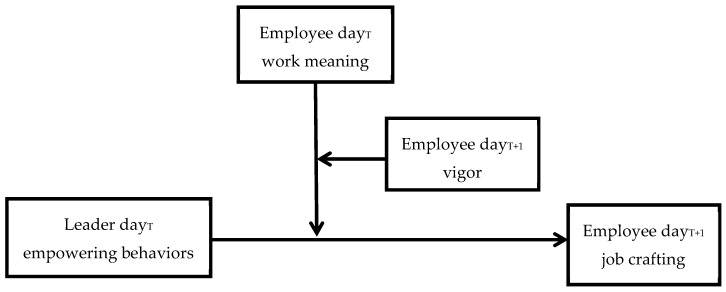
The proposed contingency model of daily leader empowering leadership and employee job crafting.

The overall research model is presented in Figure 1.

## 3. Methods

### 3.1. Procedure

Data were collected from a convenient sample of 103 participants recruited in China. The authors contacted the potential participants from personal contacts and sent the survey packages via e-mails. Before the daily survey started, participants were asked to fill in a general questionnaire measuring their demographics. Then, they were asked to complete a daily survey measuring leader empowering leadership and their own work meaning, vigor, and job crafting at the end of every workday for one week (i.e., five workdays). When all surveys were completed, participants sent back the survey packages to the authors. The daily surveys were separate excel files. From the files that participants sent back to us, we could see the time information. Participants differed in the time completing each day’s survey, but they all completed the daily survey on that particular day because we had requested them to do so and sent them reminders at the end of each workday.

### 3.2. Participants

In total, 103 out of 140 participants returned surveys with complete answers (response rate of 71%). Among the 103 participants, 47% of them were male. The mean age was 27.55 years (SD = 3.85). About 70% of them had a bachelor’s degree or above. On average, job tenure was 26.59 months (SD = 17.19). Participants were all general employees and came from 55 companies mainly from construction industry (25%), IT (21%), financial industry (15%), medical service (12%), public service (9%), real estate industry (6%), and management consulting service (6%). Most of the participants were HR managers, consultants, engineers, and programmers.

### 3.3. Measures

Leader daily empowering behaviors were measured with eight items adapted from Zhang and Bartol [32] by referring to the specific day. Each of the four dimensions of empowering leadership was assessed with two items. Here are examples: “Today, my supervisor solicited my opinion on decisions that may affect me” (consulting); “Today, my supervisor allowed me to do the job in my way” (delegating); “Today, my supervisor believed that I could handle challenging tasks” (enabling); “Today, my supervisor helped me to understand how my objectives and goals relate to that of the company” (informing). All items were scored on a five-point scale, ranging from 1 (“not true at all”) to 5 (“totally true”). The average Cronbach’s alpha was 0.87.

Employee daily job crafting was assessed with three multi-item subscales developed by Petrou et al. [23] that measured day-level seeking resources (e.g., “Today, I have asked my supervisor for advice”, four items; the average Cronbach’s alpha was 0.77), day-level seeking challenges (e.g., “Today, I have asked for more responsibilities”, three items; the average Cronbach’s alpha was 0.82), and day-level reducing demands (e.g., “Today, I have made sure that my work is cognitively less intense”, three items; the average Cronbach’s alpha was 0.69). Responses were given on a 5-point scale ranging from 1 (“not true at all”) to 5 (“totally true”). 

Employee daily work meaning was assessed with two items adapted from Hackman and Oldham [40] by referring to the specific day. The two items are “Today, the work I do has been very important to me”; “Today, my work activities have been personally meaningful to me”. Responses were given on a 5-point scale ranging from 1 (“not true at all”) to 5 (“totally true”). The average Cronbach’s alpha was 0.81.

Employee daily vigor was measured with two items adapted from Schaufeli et al. [15] by referring to the specific day. The two items are “Today, when I got up in the morning, I felt like going to work”; “Today, at my work, I felt bursting with energy”. Responses were given on a 5-point scale ranging from 1 (“not true at all”) to 5 (“totally true”). The average Cronbach’s alpha was 0.81.

## 4. Results

### 4.1. Preliminary Results

Table 1 presents the means, standard deviations and correlations among the study variables. To confirm the distinctiveness of the variables involved in the current research, we conducted multilevel confirmative factor analysis (MCFA), which included daily factors of empowering leadership with four dimensions (indicators), work meaning with two items (indicators), vigor with two items (indicators), and job crafting with three dimensions (indicators). We used RMSEA (root mean square error of approximation), CFI (comparative fit index), TLI (Tucker–Lewis index), and SRMR to evaluate the model fit. According to Hu and Bentler [41], an RMSEA smaller than 0.06, a CFI and TLI larger than 0.95, and an SRMR smaller than 0.08 indicate relatively good model–data fit in general. Results revealed that the 4-factor model had good fit (χ^2^ = 105.98, *df* = 76, *p* < 0.05, RMSEA = 0.03, CFI = 0.97, TLI = 0.95, SRMR_within_ =0.03, SRMR_between_ = 0.06). Alternative models such as a 3-factor model which combined empowering leadership and work meaning (χ^2^ = 267.72, *df* = 82, *p* < 0.01, RMSEA = 0.07, CFI = 0.83, TLI = 0.77, SRMR_within_ = 0.06, SRMR_between_ = 0.07) fit the data worse than the 4-factor model. 

### 4.2. Testing the Hypotheses 

We used multilevel modeling to test the hypotheses with the Mplus 7.4 software (Muthen & Muthen, Los Angeles, CA, USA) [42]. To test hypothesized moderation effects, we estimated a model including day_T_ (a certain day) leader empowering behavior, day_T_ employee work meaning, and day_T+1_ (next day after the certain day) employee vigor. As these variables are all at day-level, following suggestions by previous studies [43] we person-centered them to control for the influence of stable individual differences. We used day_T+1_ vigor instead of day_T_ vigor because vigor seems to fluctuate heavily from day to day. When predicting one’s job crafting on a certain day, it would make more sense to consider the level of vigor on that particular day. Unstandardized coefficient estimates are presented in Table 2. The interaction of leader day_T_ empowering behaviors and employee day_T_ work meaning was not significant for employee day_T+1_ seeking resources (γ = −0.31, t = −1.76, *p* > 0.05), daily seeking challenges (γ = −0.22, t = −1.17, *p* > 0.05), and daily reducing demands (γ = −0.31, t = −1.74, *p* > 0.05). Hypothesis 1 was thus not supported. The interaction of leader day_T_ empowering behaviors and employee day_T+1_ vigor was not significant for employee day_T+1_ seeking resources (γ = 0.11, t = 0.85, *p* > 0.05), daily seeking challenges (γ = 0.10, t = 0.76, *p* > 0.05), and daily reducing demands (γ = 0.11, t = 0.78, *p* > 0.05). Hypothesis 2 was also not supported.

**Figure 2:**
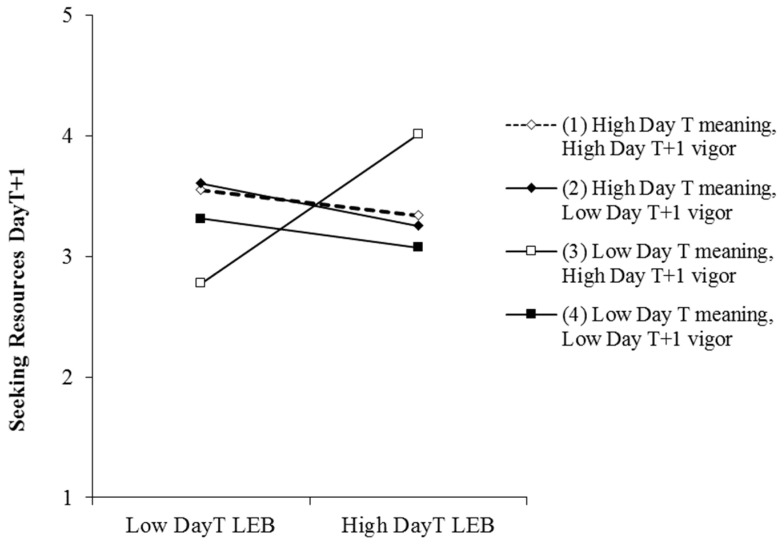
Moderating effect of employee day_T_ work meaning and day_T+1_ vigor on the relationship between day_T_ leader empowering behaviors (LEB) and employee day_T+1_ seeking resources.

**Figure 3:**
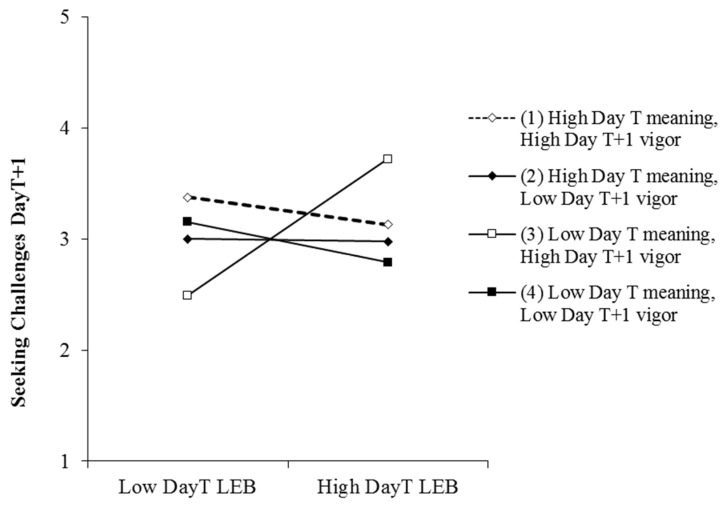
Moderating effect of employee day_T_ work meaning and day_T+1_ vigor on the relationship between day_T_ leader empowering behaviors (LEB) and employee day_T+1_ seeking challenge.

**Figure 4:**
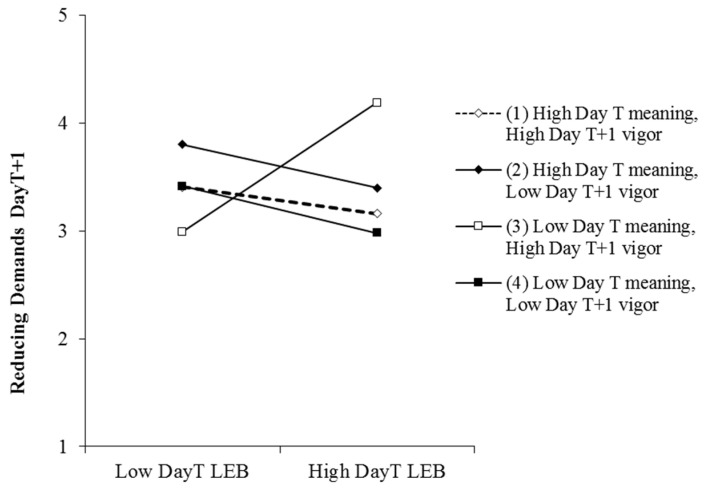
Moderating effect of employee day_T_ work meaning and day_T+1_ vigor on the relationship between day_T_ leader empowering behaviors (LEB) and employee day_T+1_ reducing demands

The three-way interaction of day_T_ empowering behaviors, employee day_T_ work meaning, and employee day_T+1_ vigor was significant for employee day_T+1_ seeking resources (γ = −0.42, t = −1.84, *p* < 0.1), daily seeking challenges (γ = −0.58, t = −2.68, *p* < 0.01), and daily reducing demands (γ = −0.47, t = −2.40, *p* < 0.05). We summarized the simple slope results in Table 3 and plotted the moderations in Figure 2, Figure 3 and Figure 4. Table 3 showed that at low levels of meaning and high levels of vigor, day_T_ empowering leadership was significantly positively related to day_T+1_ seeking resources (b = 1.02, *p* < 0.05), seeking challenges (b = 1.03, *p* < 0.01), and reducing demands (b = 0.98, *p* < 0.01). We further analyzed the differences in simple slopes. For the three dimensions of job crafting, the simple slope of the condition of low work meaning and high vigor is significantly higher than any other conditions. This means empowering leadership has the strongest relationship with job crafting when work meaning is low and vigor is high, which can be seen from Figure 2, Figure 3 and Figure 4. These findings supported Hypothesis 3. 

### 4.3. Robustness Checks

To check the robustness of our results, we included controls such as employee education level, gender, job types, and occupational sectors, as these variables may play a role in influencing to what extent leaders empower their employees. However, including these controls did not affect the significance of the hypothesized relationships. These results are available upon request.

## 5. Discussion

The present study took a contingency perspective on the relationship between empowering leadership and job crafting on a daily basis. We proposed three hypotheses: work meaning negatively moderated (H1), vigor positively moderated (H2), and work meaning and vigor (H3) jointly moderated the relationships between daily empowering leadership and three forms of daily job crafting. Our findings found evidence only for H3, namely, a three-way interaction of daily empowering leadership, work meaning, and vigor on daily job crafting. That is, daily empowering leadership resulted in a highest level of next-day job crafting when daily work meaning was low and next-day vigor was high. These results suggest that only under conditions of low work meaning (e.g., “reason to”) and high vigor (“energized to”) can empowering leadership (“can do”) promote employee job crafting on a daily basis. These findings suggest that the three motives do not work independently. Rather, all of them are necessary and need to be considered simultaneously in order to trigger job crafting. We discuss the theoretical and practical implications of our findings below. 

### 5.1. Theoretical Implications for the Literature 

Our study contributed to the literature in several ways. First, our findings challenged the notion that the effect of empowering leadership is universal in promoting employee proactive behaviors. Our study showed that empowering leadership alone did not increase employees’ job crafting behaviors. Although empowering leaders may enable employees to work more efficiently by engaging in efforts such as delegating authority and showing confidence, it may not be sufficient for the occurrence of job crafting. After all, job crafting is self-initiated and primarily driven by personal psychological needs [2]. This finding on empowering leadership and job crafting also corroborates with previous research on leadership as an antecedent of job crafting [3,12].

Second, our findings add to the growing literature on leadership and job crafting by providing a contingency perspective where external leadership forces interact with employees’ meaningful job experiences and positive affective state in driving self-initiated redesign actions. To our knowledge, few empirical studies have examined moderators of the relationship between empowering leadership and job crafting. One exception is the study by Thun and Bakker [11] which found that employee optimism strengthened the association between empowering leadership and (expansion) job crafting, suggesting that the effect of empowering leadership on job crafting is contingent on employees’ personality characteristics. Employee job crafting is a self-initiated job redesign behavior to achieve more personal meaning out of work. Our findings suggest that how employees experience their jobs (low versus high meaning) and whether they have energy at work are important factors to consider when leaders use empowerment to promote their job crafting.

Third, our study also extended the model of proactive motivation. Although it is proposed that proactive behavior is driven by the three motives, it is unclear that how they jointly predict proactive behavior [44]. There are at least three possible patterns. First, the three motives can independently predict proactive behavior. That is, each motive makes unique contributions to proactive behavior beyond the other motives. Second, the three motives can strengthen one another in predicting proactive behavior. Namely, the effect of one motive is augmented by another one. Third, only if three motives are met simultaneously can proactive behavior take place. Our results seemed to lend more support for the third possibility, as job crafting is at highest level when there is high empowering leadership (enabling “can do”), low work meaning (“reason to”), and high vigor (“energized to”). Any of the other combinations of different levels of empowering leadership, work meaning, and vigor did not reach a significant increase in employee job crafting. Consistently, a recent study by Wang, Chen and Lu [45] also found support for the joint effect of three different motives for job crafting.

### 5.2. Practical Implications, Limitations, and Future Directions

Our study has positive implications for practice. There is growing evidence showing that job crafting can bring positive work outcomes. Therefore, identifying antecedents of job crafting has attracted attention of both researchers and organizational managers. Our study showed that leader daily empowerment may simulate more job crafting among employees particularly when employees have low work meaning and high vigor. In other words, when leaders engage in empowering efforts, they should consider employees’ meaningful job experiences and their energy on a daily basis. To promote job crafting, they shall use more empowerment for employees who experience their jobs less meaningfully and who feel energized at work. 

Several limitations to this study should be noted. First, we recommend that future research apply the notion of joint motives to other forms of employee proactive behavior such as voice and taking charge. Different proactive behaviors may require specific manifestation of three motives. For example, having low work meaning is a relevant reason for one to craft the job as job crafting is a way to find more meanings, but low work meaning may not be motivating for one to engage in voice or taking charge. Second, the relationship may be inflated by common method variance since all the variables were self-reported. Yet, some of the focal variables were used from measurements on day_T_, while others on day_T+1_, which may rule out this potential problem to some extent. Third, we focused our model on the day level. It may be interesting for future research to conduct a parallel examination of the antecedents of job crafting at both person and day levels. Fourth, we used a convenient sample as the participants were approached by the snowball sampling method. Our sample was thus not representative, and caution should be taken when generalizing our findings to different groups of samples. Last, our hypothesized model mainly derived from Western theories was tested in a Chinese context. It should be noted that only the three-way interaction (i.e., H3) was supported in our study. Previous studies have revealed the effectiveness of empowering leadership in a Chinese context [32]. Additionally, individual job crafting occurs also in collectivistic cultures like China [46]. Nevertheless, we suggest that future research in other cultural contexts is needed to verify the generalizability of our findings.

## 6. Conclusions

In this paper, we aimed to develop and test a contingent model of empowering leadership and job crafting on a daily basis. Our findings based on a daily dairy study suggest that empowering leadership, work meaning, and vigor jointly predict job crafting. Specifically, empowering leadership is associated with highest levels of job crafting when work meaning is low, and vigor is high. Our findings go beyond the proactive motivation model by providing a more nuanced understanding of how drivers of employee proactive behavior interact with one another.

## Figures and Tables

**Table 1:** Means, standard deviations, and correlations among study variables.

Variables	Mean	SD	1	2	3	4	5	6
Day-level								
Leader daily empowering behaviors	3.27	0.61	1					
Employee daily work meaning	3.52	0.76	0.49 **	1				
Employee daily vigor	3.22	0.84	0.49 **	0.42 **	1			
Employee daily seeking resources	3.30	0.71	0.55 **	0.45 **	0.43 **	1		
Employee daily seeking challenges	3.05	0.81	0.51 **	0.42 **	0.43 **	0.67 **	1	
Employee daily reducing demands	3.35	0.71	0.44 **	0.35 **	0.32 **	0.59 **	0.52 **	1

Note. 103 persons, 5 days, and 515 occasions. ** *p* < 0.01.

**Table 2:** The multilevel regression models for testing the hypotheses.

Variables	Employee Day_T+1_ Seeking Resources (γ)				Employee Day_T+1_ Seeking Challenges (γ)				Employee Day_T__+1_ Reducing Demands (γ)			
	Model 1	Model 2	Model 3	Model 4	Model 1	Model 2	Model 3	Model 4	Model 1	Model 2	Model 3	Model 4
Intercept	3.35 ***	3.36 ***	3.35 ***	3.36 ***	3.07 *	3.08 ***	3.07 ***	3.08 ***	3.40 ***	3.41 ***	3.40 ***	3.42 ***
Employee day_T_ seeking resources	−0.13 *	−0.14 **	−0.13 **	−0.14 **	--	--	--	--	--	--	--	--
Employee day_T_ seeking challenges	--	--	--	--	−0.09	−0.09	−0.09	−0.10	--	--	--	--
Employee day_T_ reducing demands	--	--	--	--	--	--	--	--	−0.20 **	−0.20 **	−0.20 **	−0.20 **
Leader day_T_ empowering behaviors (LDEB)	0.04	0.03	0.02	0.09	0.05	0.04	0.04	0.13	−0.05	−0.04	−0.05	0.02
Employee day_T_ meaning	--	0.08	0.06	0.09	--	0.04	0.03	0.05	--	0.02	−0.00	0.03
Employee day_T+1_ vigor	--	0.04	0.05	0.06	--	0.09 *	0.10 *	0.12 *	--	-0.01	−0.00	0.02
LDEB × Employee day_T_ meaning	--	-0.31	--	−0.42 *	--	−0.22	--	−0.30	--	-0.31	--	−0.38
LDEB × Employee day_T+1_ vigor	--	--	0.11	0.40	--	--	0.10	0.36	--	--	0.11	0.44 **
Employee day_T_ meaning × Day_T+1_ vigor	--	--	--	−0.07	--	--	--	0.05	--	--	--	−0.27 **
LDEB × Employee day_T_ meaning × Day_T+1_ vigor	--	--	--	−0.42 (*p* = 0.07)	--	--	--	−0.58 **	--	--	--	−0.47 *

Note. N = 103 employees, N = 515 occasions. * *p* < 0.05, ** *p* < 0.01, *** *p* < 0.001.

**Table 3:** Conditional effect of leader day_T_ empowering behaviors on employee day_T+1_ job crafting at values of the moderators.

Moderators		Day_T+1_ Seeking Resources			Day_T+1_ Seeking Challenges			Day_T+1_ Reducing Demands		
Day_T_ Meaning	Day_T+1_ Vigor	Estimate	*p*	SE	Estimate	*p*	SE	Estimate	*p*	SE
Low	Low	−0.20	0.44	0.25	−0.31	0.29	0.29	−0.36	0.15	0.25
Low	High	1.02	0.02	0.44	1.03	0.01	0.38	0.98	0.00	0.30
High	Low	−0.29	0.34	0.30	−0.03	0.92	0.33	−0.33	0.32	0.33
High	High	−0.18	0.17	0.13	−0.18	0.10	0.11	−0.21	0.08	0.12

Note. The analyses of differences in simple slopes showed that for any dimensions of job crafting, the simple slope of the condition of low work meaning and high vigor was significantly higher than any other conditions.
